# Engineering the human blood-brain barrier in vitro

**DOI:** 10.1186/s13036-017-0076-1

**Published:** 2017-12-04

**Authors:** John J. Jamieson, Peter C. Searson, Sharon Gerecht

**Affiliations:** 1 Department of Chemical and Biomolecular Engineering, Johns Hopkins University, 3400 North Charles Street, Baltimore, MD 21218 USA; 2 Institute for Nanobiotechnology, 100 Croft Hall, Johns Hopkins University, 3400 North Charles Street, Baltimore, MD 21218 USA; 3 Department of Materials Science and Engineering, Johns Hopkins University, 3400 North Charles Street, Baltimore, MD 21218 USA

**Keywords:** blood-brain barrier, neurovascular unit, in vitro modeling, induced pluripotent stem cells

## Abstract

The blood-brain barrier (BBB) is the interface between the vasculature and the brain, regulating molecular and cellular transport into the brain. Endothelial cells (ECs) that form the capillary walls constitute the physical barrier but are dependent on interactions with other cell types. In vitro models are widely used in BBB research for mechanistic studies and drug screening. Current models have both biological and technical limitations. Here we review recent advances in stem cell engineering that have been utilized to create innovative platforms to replicate key features of the BBB. The development of human in vitro models is envisioned to enable new mechanistic investigations of BBB transport in central nervous system diseases.

## Background

Neurons in the brain require a chemically stable environment, insulated from fluctuations in blood components in circulation [1–3]. The BBB maintains homeostasis by regulating molecular transport between the cardiovascular system and the central nervous system (CNS), and also protects the CNS by restricting the entry of xenobiotics and immune cells that could cause inflammation [4].

The physical integrity of the barrier is derived from the endothelial cells (ECs) that line the brain microvasculature and tightly control paracellular and transcellular transport [2]. Paracellular transport is restricted by tight junctions (TJs) that stitch together adjacent ECs, while transcellular transport is regulated by a combination of specialized transporters and efflux pumps. Transporters supply essential nutrients to the brain, while efflux pumps counter the passive entry of small molecules, including many toxins, but also many potential therapeutics. ECs in the CNS are supported structurally and functionally by pericytes, basement membrane, and astrocytes [5]. Interactions between these components contribute to the development and maintenance of the healthy BBB [6–8], although the relative contributions of each component and the specific mechanisms by which these processes occur is an area of active research, which will be discussed in more detail later.

The intact BBB constitutes a major roadblock for drug delivery, as 98% of small molecules are unable to enter the brain [9]. Strategies to enhance delivery have included either modifications to therapeutic agents, exploiting receptor-mediated transport systems [10], or temporary disruption of the BBB,for example by osmotic agents [11] or focused ultrasound (FUS) [12]. Approaches to take advantage of receptor-mediated transport (RMT) systems, including the Transferrin receptor (TfR), have had some preclinical success in delivering protein therapeutics [13]. Developing new CNS therapies or delivery techniques requires a detailed understanding of the mechanisms of BBB transport, as well as extensive testing and optimization in model systems.

The sequence of steps in drug development generally include in silico modeling, testing in in vitro models, studies in animal models, and human trials. Animal models have been shown to lack consistent predictive value for humans, with 50% of results not translating into human responses [14]. Cross-species differences in the BBB limit, and in some cases prohibit, the applicability of animal models. For example, recent studies compared the expression levels of TJ proteins and transporters expressed by various mammalian species used in preclinical trials [15–17]. The results of several of these studies have recently been tabulated (see Table 1 in [18]). Notable findings included differences in the expression of the efflux transporters Breast Cancer Resistance Protein (BCRP) and P-glycoprotein (P-gp) (1.85-fold higher and 2.33-fold lower, respectively, in humans as compared with mice), as well as a 5-fold reduction in L-type amino acid transporter-1 (LAT-1) in humans as compared with mice [15]. Lastly, several transporters reported in the rodent BBB were not detected at all in the human BBB [15].

**Table 1:** Sources of cells used to replicate BMEC function

Barrier Cell Source	Origin (cell line)	TEER (Ω cm^2^)	Advantages	Disadvantages	References
Immortalized	• canine kidney epithelial (MDCK) • human colon adenocarcinoma epithelial (Caco-2) • mouse BMEC (BEnd.3) • rat BMEC (RBE4) • human BMEC (hCMEC/D3 and hBMEC)	40–315 compiled in [96]	• stable over numerous passages • commercially available • can be transfected to express human efflux pumps (MDCK)	• incomplete tight junctions • poor barrier function	[53, 99, 123]
Primary	Mouse, rat, porcine, bovine, human BMECs	130–2200 compiled in [96]	• close initial resemblance to in vivo conditions	• tedious purification with low yields and batch variability • senesce after few passages • difficult to obtain healthy tissue (human)	[98, 124–126]
PSC-derived	Mouse or human iPSC or ESC	250–5350 [19, 101, 102]	• renewable source • patient specific • physiological TEER	• require differentiation and thorough characterization	[20, 101, 102, 112]

The differential expression of transport proteins across mammalian species can affect drug uptake, leading to potentially unpredictable clinical results when moving towards human trials. One study noted that the common marmoset is a better predictor of human BBB transport than either Sprague Dawley or Wistar rat models, as most of the marmoset transporter proteins tested were within two-fold of human expression levels [17]. However, some BBB disorders cannot be studied in animal models, such as forms of meningitis caused by human-specific pathogens [19]. These limitations highlight the need for a human in vitro model to study BBB dysfunction in CNS disease progression and to help predict drug transport across the human BBB in vivo.

The development of human BBB models has been accelerated by recent advances in stem cell biology. Human induced pluripotent stem cells (hiPSCs) can be used to generate each of the cell types contributing to the BBB [20–24]. Importantly, hiPSCs can be derived from patients, allowing for the generation of both diseased and healthy versions of each cell type, which can be used to identify cell type-specific defects responsible for BBB dysfunction in disease progression. Two recent studies each used this approach to identify defects in brain microvascular endothelial cells (BMECs) derived from patients with Huntington’s Disease [25] and Allan-Herndon-Dudley syndrome [26]. Though not a replacement for animal models, a fully human in vitro model could complement animal models by providing a controlled, high-throughput system free from cross-species differences.

The goal of this review is to define the challenges associated with recapitulating the human BBB in in vitro models and to provide perspective on future model development. First, the BBB’s salient features will be outlined and its cellular components reviewed. Then, design criteria for developing a dynamic, multicellular, human BBB model will be established and recent progress towards these goals will be reviewed.

### The BBB and the neurovascular unit

**Fig. 1:**
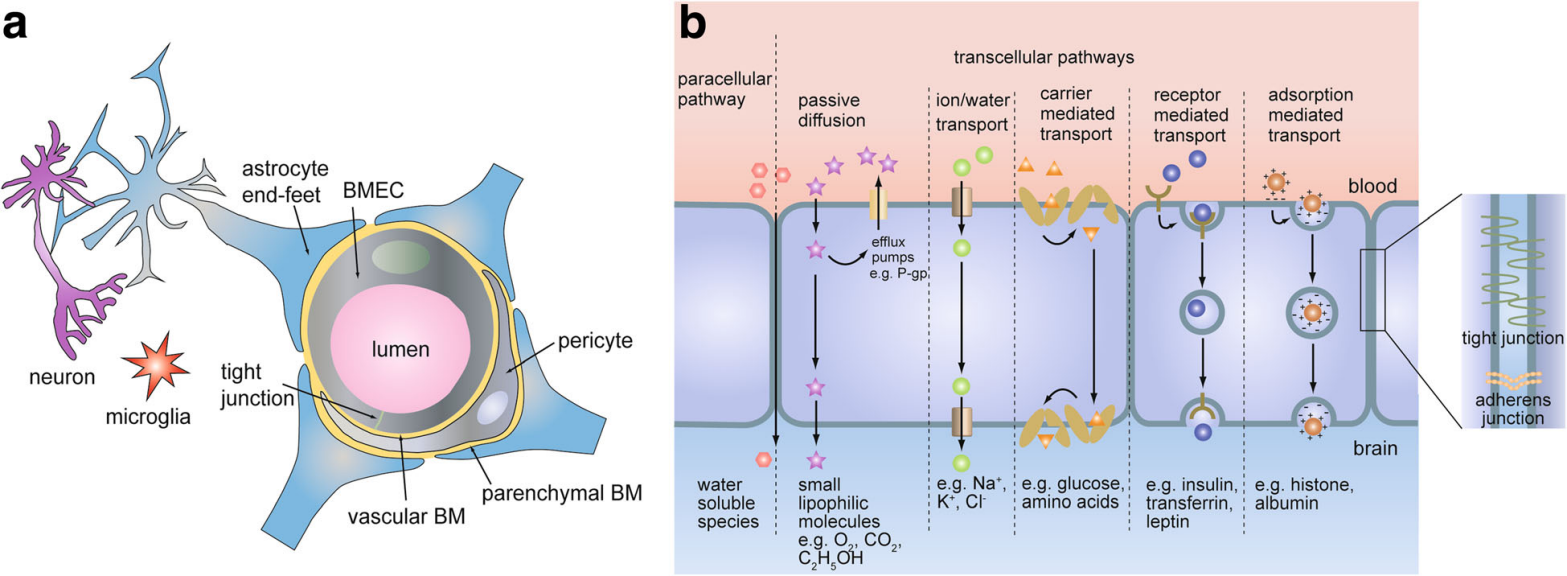
Structure and function of the BBB. (a) Schematic representation of the cell types that form the NVU. (b) Paracellular and transcellular pathways of molecular transport across the BBB

The majority of transport between the vascular system and the brain occurs in brain microvessels, as these comprise approximately 95% of the area between the brain and the vascular system [27]. The BBB includes BMECs, basement membrane, pericytes, and astrocyte end-feet. (Fig. 1a). These components physically and biochemically interact in order to maintain barrier function. While BMECs are the cells directly responsible for restricting and regulating transport, the surrounding layer of basement membrane embedded with pericytes provides structural support and depots for molecular signals that regulate EC function. The microvessels are surrounded by protrusions from astrocytes that terminate in end-feet, which play important roles in maintaining homeostasis [28] and regulating blood flow to regions of high neuronal activity [29]. As a result of the coordinated interactions between BMECs, pericytes, astrocytes, neurons, and CNS immune cells, this group is often collectively referred to as the neurovascular unit (NVU).

### Brain microvascular endothelial cells (BMECs)

BMECs are morphologically, biochemically, and functionally distinct from non-brain ECs. In addition to expressing conventional adherens junction (AJ) proteins such as VE-cadherin and PECAM, adjacent BMECs are stitched together by TJs, reducing paracellular transport between neighboring cells [30] (Fig. 1b). TJs are formed by interactions between transmembrane proteins including claudins, occludins, and junctional adhesion molecules (JAMs), which are linked to the cytoskeleton through TJ adapter proteins such as zonula occludens-1 (ZO-1) and cingulin. BMECs also lack fenestrations and exhibit reduced transcytosis relative to non-brain ECs [31, 32].

Although TJs and reduced transcytosis indiscriminately restrict the transport of ions and molecules, the selectivity of the BBB is imparted by polarized expression of several classes of nutrient transporters and efflux pumps (Fig. 1b) that have been reviewed elsewhere [2]. Here we highlight several systems that could be examined in order to demonstrate BMEC polarization and transport in an in vitro system.

One class of polarized transporters is the solute carrier (SLC) family, which enables the passive transport of polar nutrients essential to CNS function, such as glucose (Glut-1) and amino acids (LAT-1, among others). Differential expression of these transporters on the luminal and abluminal membranes of BMECs regulate CNS nutrient uptake and waste removal. Another class of polarized transporters is the efflux pumps of the ATP-binding cassette (ABC) superfamily. Small lipophilic molecules, which would typically diffuse through non-brain ECs, are actively effluxed back to the blood by BMECs. Notable efflux pumps include P-gp, BCRP, and Multidrug Resistance-associated Proteins (MRPs) [2]. Efflux pumps often work in tandem with metabolizing enzymes, together breaking down and pumping out potentially toxic substances, including many conventional therapeutics [27].

For larger molecules and proteins, such as transferrin, insulin, and IgG, transport is usually receptor-mediated (RMT) or adsorptive-mediated (AMT) [2] (Fig. 1b). Efforts to deliver therapeutics through these pathways are informed by studies into the kinetics of receptor internalization and recycling, and the effects of ligand design on these parameters [9]. Many of the receptors involved in RMT are poorly characterized, bind multiple ligands, and exhibit multiple functions. Advances in the understanding of these mechanisms and their regulation could result in improved methods of drug delivery to the CNS.

The unique properties of BMECs are induced by the surrounding neuroectodermal environment during development, although the exact mechanisms responsible remain poorly understood [33]. Initial evidence from quail-chick chimera transplantation studies showed that non-CNS tissue grafted to the brain could develop BBB characteristics, while CNS-tissue grafted to non-CNS regions could not [33]. Recent studies have identified several pathways believed to be critical to BBB induction and maintenance, including hedgehog (Hh) [6] and canonical Wnt signaling [34, 35]. The importance of Wnt/ β-catenin signaling was further demonstrated by β-catenin-deficient mouse embryos that exhibited widespread vascular defects in the CNS while peripheral vessel formation was unaffected [36].

In addition to molecular signaling, many important characteristics of BMECs may be induced by hemodynamic forces, including shear stress (approximately 5–20 dyne cm^−2^ in capillaries [3, 37, 38]) Shear stress has been shown to activate mechanotransduction pathways in ECs influencing the expression of genes regulating functional behavior including proliferation, migration, and inflammation [38–40]. While these effects have been broadly documented across ECs of other organs, the response of BMECs to shear stress appears unique. While human umbilical vein endothelial cells (HUVECs) elongate in the direction of flow, BMECs instead remain rounded [41, 42]. Other physical characteristics of capillaries, such as the degree of vessel curvature, have been shown to elicit elongation and alignment from HUVECs but not from immortalized BMECs, providing further evidence of their unique phenotype [43]. Despite these advances, many details of the phenotype of BMECs remain to be resolved.

### Pericytes

Pericytes play an important role in vascular development, as they are recruited to stabilize nascent vessels and promote vascular maturation [7, 44]. Pericyte recruitment is driven in part by EC expression of platelet derived growth factor (PDGF) [44]. As pericytes are found throughout the body, their role in BBB induction and maintenance was mostly overlooked until several groups demonstrated their importance in vivo [8, 45, 46]. Pericyte-deficient mice display abnormal, leaky vasculature with an increased rate of transcytosis [8, 45]. Additionally, improper localization of aquaporin 4 (Aqp4) in astrocytes in pericyte-deficient mice suggests that pericytes influence the polarization of astrocyte end-feet, and mediate the attachment of end-feet to CNS vasculature [45], although a separate study observed normal astrocyte attachment in pericyte-deficient mice [46]. Furthermore, as pericyte-deficient mice aged, they experienced progressive BBB breakdown and cognitive impairment, demonstrating that pericyte loss can precede neurodegenerative effects [46]. These in vivo studies have collectively shown that pericytes may coordinate NVU assembly and play a key role in BBB induction and maintenance. They also suggest that pericytes predominantly exert this effect through the inhibition of transcytosis, rather than the induction of BBB-specific transporters or TJ formation [8, 45, 47].

The effects of pericytes on BBB function have also been studied in vitro. Pericytes co-cultured with various sources of BMECs have been found to increase barrier function, albeit to greatly differing extents [48–53], and have also been found to interfere with barrier function under certain conditions [54, 55]. The mechanisms by which pericytes regulate BMECs are not fully understood, however, PDGF, VEGF, TGF-β, and Notch pathways are implicated (reviewed in [44]). Although transwell models have been used to study paracrine signaling pathways, physical connections between pericytes and ECs have also been reported to play important roles in vivo, transmitting mechanical forces through adhesion plaques [56], and transporting signaling molecules directly through gap junctions [57].

An important question regarding BBB induction by pericytes is how this interaction is localized to the CNS, as pericytes are found throughout the body. Interestingly, while most pericytes are believed to be of mesodermal origin, some studies have suggested that CNS pericytes derive from the neural crest [58–61], and thus may be functionally distinct from peripheral pericytes [8]. Additionally, the increased ratio of pericytes to ECs found in the brain (1:3–1:1, as compared to 1:100 in skeletal muscle) further support an important role for pericytes in BBB function, as increased pericyte coverage throughout the body has been correlated with increased vessel tightness [62].

### Basement membrane

The basement membrane (BM) is a thin layer of extracellular matrix (ECM) surrounding the microvasculature. The BM interacts with cells through physical and biomolecular pathways to mediate cell attachment and differentiation. There are two layers of BM, with distinct composition, referred to as the vascular (or endothelial) BM and the parenchymal BM, located abluminal to the ECs and PCs, respectively [63]. In capillaries, these membranes are fused, while in post-capillary venules, they are separated by a perivascular gap, known as the Virchow-Robin space, a key location for leukocyte trafficking and immune cell regulation [4, 19, 64].

The BM is composed of highly cross-linked networks of structural and specialized proteins collectively secreted by endothelial cells, pericytes, and astrocytes [65]. Type IV collagen and laminin are each capable of self-assembling networks, which are then interconnected by nidogens and heparan sulfate proteoglycans, such as perlecan [63]. There is a rich complexity in BM composition, as over 50 other glycoproteins have been found in varying quantities as minor components. Furthermore, multiple isoforms of each BM component exist and many exhibit distinct binding profiles [63]. These specialized BM proteins bind transmembrane proteins including integrins, anchoring ECs and pericytes in place, and transducing signals to the actin cytoskeleton which regulate cellular behaviors and promote quiescence [65].

The functions of various BM proteins have been informed in part by studies on knockout mice. Recent knockout studies revealed that astrocyte-derived laminin-211 is critical for maintaining BBB integrity [66, 67]. Interestingly, this effect was reported to act through the regulation of pericyte differentiation [66], in agreement with an earlier in vitro study which suggested that α-SMA^-^ and α-SMA^+^ pericytes raise and lower TEER, respectively [55]. This demonstrates the ability of BM compositional changes to serve as an intermediary in BBB cell-cell signaling and regulation.

The BM can become altered by protease activity in response to inflammation or disease. Cytokines produced by astrocytes and pericytes, such as interleukin (IL)-6, can trigger EC release and activation of matrix metalloproteinases (MMPs), which are capable of degrading ECM components [68]. MMP-2 and MMP-9 can proteolyze collagen IV, elastin, and fibronectin, while MMP-2 can additionally cleave laminin [69]. Loss of BM may lead to BBB dysfunction, as BM disruption has been shown to promote cytoskeletal alterations in ECs that affect TJs [69]. BM thinning has also been observed to precede pericyte migration away from the endothelium [70] and detachment of astrocyte end-feet [4]. The various pathways by which BM modifications influence BBB function deserve further study. Relatively few in vitro studies have addressed BM interactions in the BBB, and these have generally been performed on models far more simplistic than the in vivo BM [54, 68, 71–73].

### Astrocytes

Astrocytes mediate signaling between neurons and BMECs. Astrocyte processes are terminated in end-feet that completely ensheath microvessels and capillaries in the brain [74]. A single astrocyte contacts on average five different blood vessels and four different neuronal somata, supporting the function of roughly 2 million synapses [75, 76]. This position as an intermediary allows astrocytes to coordinate key aspects of neurovascular coupling, including the regulation of blood flow to match local neuronal activity [29].

Astrocytes have been shown to induce BBB function by enhancing TJ formation, polarizing transporters, and promoting specialized enzymes [77, 78]. Numerous in vitro studies have confirmed that astrocytes secrete soluble factors, including glial-derived neurotrophic factor (GDNF), basic fibroblast growth factor (bFGF), and angiopoetin-1 (Ang-1), which have been found to increase barrier tightness [5, 77]. Astrocytes also secrete Sonic hedgehog (SHh), retinoic acid (RA), and angiotensin-converting enzyme-1 (ACE-1), which have been shown to induce the expression of junctional proteins in ECs [6, 79].

### Neurons

There are approximately 100 billion neurons in the adult brain [3], located on average, 10–20 μm away from the nearest capillary [80]. Each neuron is extensively networked to other neurons and glial cells through synapses. At synapses, electrical action potentials are transduced to molecular signals through the release of neurotransmitters, such as glutamate. This release of glutamate initiates a variety of neurovascular interactions, including the regulation of blood flow to match neural activity patterns. This appears to occur through at least two major pathways: (1) raising Ca^2+^ levels in neurons resulting in the secretion of nitric oxide, which dilates blood vessels, and (2) raising Ca^2+^ levels in astrocytes, stimulating multiple pathways, including the release of K^+^ ions to the vasculature (reviewed in [29]). The role of neurons in regulating BBB function remains poorly understood.

### Immune cells

While not a structural component of the BBB, immune cells are often included in the NVU as they have significant influence on barrier function in response to injury and disease. The two main CNS immune cell types are microglia and perivascular macrophages. Microglia are yolk-sac derived cells of myeloid lineage differentiated in the brain parenchyma during embryonic development. Immune activity of microglia is normally suppressed by electrical activity of neurons [4, 81]. However, when activated, microglia express major histocompatibility complex (MHC) Class I and II molecules and can assist perivascular macrophages as antigen presenting cells (APCs) [4, 81].

Perivascular macrophages also play an important role in regulating immune cell trafficking across the BBB, which often occurs in post-capillary venules [4]. In contrast with microglia, these cells are routinely replaced by progenitors from circulation [82], demonstrating that leukocytes can be transported across the healthy BBB.

Although the CNS is generally regarded as immune privileged in recognition of the fact that a proinflammatory T-cell response is not generated when immunogenic material is introduced to the brain parenchyma [81, 83], CNS immune cells can recruit macrophages during an innate immune response, and are able to generate a T-cell response under certain circumstances through communication with the peripheral immune system. (For reviews, see [4, 81]).

### In vitro BBB modeling

In vivo studies in the human brain are limited to non-invasive imaging, such as positron emission tomography (PET) and blood oxygen level dependent functional magnetic resonance imaging (BOLD fMRI) [84]. In vitro models, such as variations of the transwell assay, have been widely used to study BBB barrier function [27]. Reliable and reproducible sources of BMECs and supporting cell types has been a major limitation in these studies. However, hiPSCs have provided a new source of human BMECs, pericytes, and astrocytes that has enabled the study of the differentiation and development of the human BBB.

**Fig. 2:**
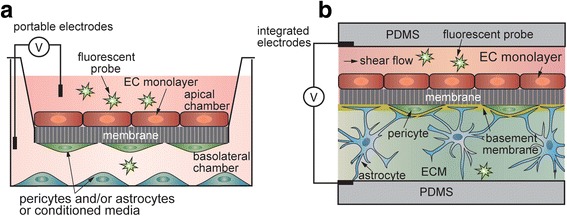
TEER and permeability measurements for assessing barrier function. (**a**) The transwell model, with an EC monolayer on the apical side of the membrane, and supporting cell types in the ‘contact’ and ‘non-contact’ positions on the underside of the membrane and in the basolateral chamber. TEER is measured between electrodes located in each compartment. Permeability is measured by introducing a solute of interest into the apical chamber and measuring the time-dependent concentration in the basolateral chamber. (**b**) A microfluidic version of the transwell model.

The transwell assay is the most widely used in vitro assay for BBB research, with applications in drug screening and in mechanistic studies of BBB regulation [27, 85–87]. In this assay, a confluent monolayer of ECs is formed on a porous membrane that separates apical and basolateral chambers (Fig. 2a). The addition of astrocytes, pericytes, and/or neurons, or media conditioned by these cells, in the basolateral chamber is often used to upregulate barrier function [48, 85]. The transport of solutes or cells from the apical to basolateral chamber can be used to determine permeability, mechanisms of transport, and the role of inflammatory cytokines, pathogens, etc. [27].

The two most commonly used parameters for quantitative assessment of barrier function are transendothelial electrical resistance (TEER) and permeability. TEER measurements can be performed in real time and are not damaging to cells [88]. In the classical transwell assay, a cell monolayer is cultured on a membrane insert, with media on each side. A voltage (or current) is applied between electrodes placed in the apical and basolateral compartments, and the impedance is calculated based on the resulting current and normalized to the surface area (Fig. 2a). While TEER values across human BMECs cannot be easily measured in vivo, TEER values across rat and frog brain ECs have been measured in the range of 1200–1900 Ω cm^2^ [89, 90]. In contrast, non-brain ECs have a TEER of about 10 Ω cm^2^ [89]. TEER values for primary BMECs are highly variable, dropping quickly after just 1–2 passages. Madine Darby Canine Kidney (MDCK) cells, the most widely used cell line in BBB research, typically exhibit TEER around 100 Ω cm^2^ [91], much lower than physiological BBB values.

Permeability (cm s^−1^) is defined as solute flux through unit area under unit concentration gradient [3, 92]. Lucifer yellow and a range of molecular weight FITC-dextrans are widely used to assess barrier function (Fig. 2). Permeability across the BBB in rodents can be measured using in situ brain perfusion, which involves administration of a drug to the carotid artery and measuring the drug concentration in the brain via radio-isotopes or LC-MS/MS [93]. Permeabilities of small molecules obtained from the transwell assay using MDCK (MDR-1 MDCK) type II cells, which have been transfected to overexpress the human P-gp efflux pump, show a reasonably good correlation (R^2^ = 0.82) with in situ perfusion studies, allowing a estimation to made for transport across the human BBB [92]. While the absolute permeabilities for a given compound will typically be higher across MDCK cells than for in situ perfusion, this correlation provides a reasonable prediction, especially if the TEER value exceeds a certain threshold (typically about 250 Ω cm^2^) [94]. Compilations of TEER and permeability data from the transwell assay using various cell types can be found in the literature [92, 95, 96]. For more information on the techniques themselves, the reader is referred to reviews concerning TEER measurement [3, 88] and permeability [3, 97].

Recapitulating the NVU with in vitro models is extremely challenging, and requires advances in many areas. The first challenge is a source of BMECs that exhibit tight junctions, low permeability, high TEER, and polarized efflux transporters. The second challenge is co-culture with other components of the NVU, including astrocytes, pericytes and BM with the correct spatial organization and biomolecular signaling. Third, models should reproduce the cylindrical geometry of brain capillaries, recapitulating the shear flow and curvature associated with brain capillaries. In the next section, we discuss sources of BMECs and other NVU cells that are used to model BBB function in vitro, then review the platforms used to configure these cells.

### Cell sources

ECs from a variety of sources have been used to model BBB function, including primary, immortalized, and PSC-derived, across a range of mammalian species (Table 1). Primary BMECs are difficult to purify and lose BBB phenotype quickly [96, 98]. Immortalized BMECs, while convenient, generally exhibit poor barrier function, making them unsuitable for applications requiring physiological TEER or permeability [96, 99]. Primary or immortalized sources of other NVU cell types (such as C8-D1A astrocytes), may suffer similar limitations, and are usually of animal origin.

An alternative to primary and immortalized BMECs is the use of cells derived from hiPSCs. hiPSCs have the potential to provide an unlimited, self-renewable, and scalable source of human BMECs for BBB research [100]. Additionally, astrocytes and pericytes can be generated from the same source of hiPSCs, enabling a fully human, syngeneic BBB model [23, 24]. Challenges to adopting hiPSC-based cellular sources include identifying and recreating conditions suitable for guiding each differentiation and demonstrating comparable functionality to cells in vivo.

hiPSC-derived BMECs have been obtained through a co-differentiation of ECs/neural cells, followed by a purification based on selective adhesion [20, 101–103]. hiPSC-derived BMECs possess localized AJs and TJs, express BBB nutrient transporters and demonstrate polarized efflux of rhodamine 123 [20, 101–103]. hiPSC-derived BMECs also exhibit physiological values of TEER [20, 101–103]. In some cases, especially with low intrinsic TEER values, co-culture with pericytes and neural progenitor cell-derived astrocytes and neurons may increase TEER [23].

hiPSC-derived pericytes have been isolated from spontaneously differentiating embryoid bodies (EBs) [104] or more recently through directed monolayer differentiation [21, 105]. These strategies seek to replicate mesoderm induction and vascular specification and result in bicellular populations of ECs and pericytes. Pericytes are isolated either by expansion in conditions that favor pericyte growth [21], or depleted of ECs based on negative selection for CD31 or VE-cad through fluorescence-activated or magnetic-activated cell sorting (FACS or MACS) [104–106]. hiPSC-derived pericytes are characterized by their expression of pericyte markers, which often include PDGFR, NG2, calponin, aSMA, CD73, CD105, CD44, and CD146 [7, 21, 107]. As a result of the limited understanding of the morphological and functional differences between pericytes in different tissues, it is difficult to establish whether pericyte differentiations can be considered brain-specific.

hiPSC-derived astrocytes have been generated by multiple groups through various embryoid body or monolayer techniques (reviewed in [108]). Typically, hiPSC-derived astrocytes are generated through an intermediate stage of neural progenitor cells (NPCs), which possess multilinage potential to form astrocytes, neurons, and oligodendrocytes. NPCs are generated by culturing hiPSCs in high concentrations of epidermal growth factor (EGF) and basic fibroblast growth factor (bFGF) [109, 110]. Extended culture of NPCs in astrocyte medium generates astrocytes characterized by the presence of GFAP and S100β [22–24].

Further elucidation of the pathways involved in BBB development and cellular response to molecular, chemical, and mechanical cues will allow researchers to develop and refine differentiations to produce cells optimal for use in human BBB models. Incorporation of multiple cell types into an in vitro BBB model must take into consideration conditions which will promote quiescence. Activated astrocytes secrete inflammatory cytokines, as well as matrix metalloprotease-9 (MMP-9) and vascular endothelial growth factor (VEGF), which can decrease barrier function. Recently, a 3D matrix composed of collagen type I, hyaluronic acid (HA), and growth factor reduced matrigel, designed to reflect the composition and mechanical properties of the brain ECM, was found to induce star-like morphology and low levels of GFAP expression typical of quiescent astrocytes [111]. Studies examining the effect of media and matrix conditions on each cell’s phenotype type are essential to replicate healthy BBB function in vitro.

### In vitro platforms

Platforms for configuring BBB cells are subject to many technical design considerations. In the context of recapitulating the complete BBB, an ideal platform would supply physiological levels of shear stress as well as facilitate the correct spatial organization of NVU components, allowing them to form realistic cell-cell junctions and basement membrane. While the transwell assay remains the most widely used platform, a number of models have sought to satisfy these other criteria. In vitro platforms have been classified and compared in Table 2.

**Table 2:** Platforms for configuring cells to replicate the BBB

Platforms for BBB modeling	Advantages	Disadvantages	References
transwell model	• replicates confluent monolayer • suitable for basic co-culture • easy measurement of TEER and permeability • does not require pumps or microfabrication	• lacks shear stress • lacks cylindrical geometry • lacks proper heterotypic cell-cell contacts	many compiled in [95, 96]
membrane-based microfluidic models	• replicates shear stress • 2D plane allows for convenient imaging	• lacks cylindrical geometry and ECM	[112–114]
matrix-containing microfluidic models	• replicates shear stress • replicates a 3D environment for embedded cells • allows for some dynamic reorganization	• matrix can pose technical challenges, including contraction • lacks cylindrical geometry	[115]
templated perfusable models	• replicates shear stress • replicates a 3D environment for embedded cells replicates cylindrical geometry	• difficult to fabricate < 20–50 μm diameter vessels • cannot measure TEER	[116, 120, 121]

Most dynamic models of the BBB extend the two-dimensional membrane-based approach by incorporating a 10 μm thick transwell membrane into a microfluidic device. Permeability measurements can be made by adding small molecules to the culture media, and TEER can be measured through the use of integrated electrodes [112–114]. These devices are designed to be improvements over the transwell assay, while remaining relatively inexpensive and high-throughput, in order to be suitable for drug permeability studies. In a variation of the membrane-based microfluidic models, an extracellular matrix can be incorporated into the channel underneath the porous membrane, allowing co-culture of other cell types in a 3D matrix [115] (Fig. 2b). Although still featuring planar geometry and a porous membrane interfering with complete cell-cell contact, these models are closer to the microenvironment of the BBB, enabling more advanced in vitro studies of drug permeability which could also examine the effect on neurons. However, recapitulating the phenotype of brain pericytes and quiescent astrocytes remains a significant challenge.

Templated perfusable models can be created using a variety of methods, including the gelation of ECM around a removable template rod [116, 117], lithography [118], 3D printing [119], and viscous fingering [120]. These systems are capable of replicating microvessel geometry and allow dynamic reorganization of co-cultured cells. Although the fabrication of these models is time-consuming, their sophistication allows researchers to examine complex interactions such as neuroinflammation [120], or visualization of drug transport across the endothelium in real time [116, 117]. Permeability has been successfully measured by quantitative fluorescent detection of molecular transport across the endothelium [116, 117, 121].

A difficult challenge facing in vitro BBB platforms is the fabrication of perfusable, capillary-dimension vascular networks. The majority of the surface area and thus transport within the BBB occurs in capillaries, which exhibit an average diameter of around 8 μm in humans [3]. Yet the smallest microvessels fabricated through any of these techniques is approximately 20–50 μm, due to the difficulties in achieving sufficiently high EC seed density in small channels without clogging [118, 122]. The prevalent approaches to overcome this issue are to stimulate capillary angiogenesis from larger microvessels, or to stimulate vasculogenesis of ECs embedded in a matrix. Non-brain capillary formation has recently been observed between adjacent microvessels in vitro. These capillaries are perfusable to fluorescent beads and maintain barrier function when perfused with fluorescent dextran [121].

A next step for in vitro BBB models is to develop perfusable brain-specific capillaries using BMECs within a matrix surrounded by physiological connections with other cells of the NVU. Tissue engineering at this scale will allow for unprecedented mimicry of BBB behavior in a controlled environment.

## Conclusions

Here, we have reviewed the components of the NVU and discussed approaches to model the BBB. In vitro BBB models can provide valuable information by serving as a high-throughput complement to animal models. Current models vary greatly with regard to cost, technical demands, recapitulated BBB aspects, and intended applications. However, there is a critical need to engineer more representative human BBB models capable of recapitulating BBB function and dysfunction. This will require integration of recent advances in stem cell technology with advances in microvessel microfabrication. The development of models that more closely resemble the human BBB will be important in gaining new insight into the structure and function of the BBB and its role in development and disease.

## Abbreviations

AMT: Adsorptive-mediated transport
BBB: Blood-brain barrier
BM: Basement membrane
BMECs: Brain microvascular endothelial cells
BOLD fMRI: blood oxygen level dependent functional magnetic resonance imaging
CNS: Central nervous system
ECM: Extracellular matrix
ECs: Endothelial cells
FUS: Focused ultrasound
hiPSCs: Human induced pluripotent stem cells
MDCK: Madine-Darby canine kidney
NVU: Neurovascular unit
PET: Positron emission topography
RMT: Receptor-mediated transport
TEER: Transendothelial electrical resistance
TJs: Tight junctions
